# First Draft Genome Sequence of *Staphylococcus condimenti* F-2^T^

**DOI:** 10.1128/genomeA.00499-16

**Published:** 2016-06-02

**Authors:** Beiwen Zheng, Xinjun Hu, Xiawei Jiang, Ang Li, Jian Yao, Lanjuan Li

**Affiliations:** a State Key Laboratory for Diagnosis and Treatment of Infectious Diseases, Collaborative Innovation Center for Diagnosis and Treatment of Infectious Diseases, The First Affiliated Hospital, College of Medicine, Zhejiang University, Hangzhou, China; b Department of Infectious Diseases, The First Affiliated Hospital, and College of Clinical Medicine of Henan University of Science and Technology, Luoyang, China

## Abstract

This report describes the draft genome sequence of *S. condimenti* strain F-2^T^ (DSM 11674), a potential starter culture. The genome assembly comprised 2,616,174 bp with 34.6% GC content. To the best of our knowledge, this is the first documentation that reports the whole-genome sequence of *S. condimenti.*

## GENOME ANNOUNCEMENT

Coagulase-negative staphylococci (CoNS) are mainly found on the skin and mucosa of humans and are regarded as less pathogenic than coagulase positive staphylococci (1 – 3). However, the widespread use of matrix-assisted laser desorption/ionization time-of-flight mass spectrometry (MALDI-TOF MS) has allowed an increasing detection rate of CoNS species from clinical samples (4). CoNS represent the most common cause of catheter-related bloodstream infections, prosthetic valve endocarditis, and prosthetic joint infections (5). *Staphylococcus condimenti* is a member of the CoNS. *S. condimenti* strain F-2^T^ (= DSM 11674) is the type strain, which was originally isolated from soy sauce mash (6). It is usually associated with food and used in starter cultures (7, 8). Although rare, *S. condimenti* also has been reported as a human pathogen causing catheter-related bacteremia (5). However, little is known about genetic determinants that contribute to its virulence and survival.

Here, we present the genome sequence of *S. condimenti* strain F-2^T^, as part of the CoNS whole-genome sequencing program initiated by our lab. Genomic DNA was prepared using the PureLink Genomic DNA minikit (Invitrogen, USA) (9). Libraries were prepared for sequencing with Nextera DNA kits (Illumina) and were sequenced on the Illumina HiSeq 2000 system, according to standard Illumina protocols. The raw reads were trimmed and assembled as previously described (10). Predicted genes were identified using Glimmer (11). tRNAscan-SE (12) was used to find tRNA genes, whereas ribosomal RNAs were found by using RNAmmer (13). The draft genome was annotated using the RAST server (14). All annotated genes were then classified based on their COG classes (15). Putative phage sequences were identified by PHAST (16). CRISPRFinder was used to screen for the presence of CRISPR arrays (17).

The draft genome sequence of *S. condimenti* strain F-2^T^ comprises 80 contigs with a total length of 2,616,174 bp and a GC content of 34.6%. It is covered at a 165-fold depth with an *N*_50_ of 136,415 bp. The shotgun sequence encodes 2,547 predicted genes. These scaffolds also contain 60 tRNAs and 14 incomplete rRNAs. CRISPRFinder revealed 2 CRISPR arrays.

Coding sequences were analyzed to detect toxin genes by using VirulenceFinder (http://cge.cbs.dtu.dk/services/VirulenceFinder), which revealed that strain F-2^T^ possesses a varying repertoire of putative virulence factors involved in adherence, such as extracellular fibronectin binding protein, fibronectin binding protein, elastin binding protein, and autolysin.

Prediction of putative phage elements revealed the presence of an intact prophage region together with one questionable prophage region. It is clear that prophages are directly associated with the virulence in *Staphylococcus aureus* (18). Proteins were also compared with the antibiotic resistance gene database (19), and we found two genes encoding proteins that belong to the β-lactamase family. Two putative CRISPR repeat regions were detected in the genome. The origin of the CRISPR systems in *S. condimenti* still remains unknown; however, the propagation of CRISPR has been proposed to occur through horizontal gene transfer by conjugation (20).

The genome sequence of *S. condimenti* F-2^T^ will contribute to easier genetic manipulation of this strain and will enable further studies in the future.

### Nucleotide sequence accession numbers.

The whole-genome shotgun project of *S. condimenti* DSM 11674 has been deposited at DDBJ/EMBL/GenBank under the accession number LAQN00000000. The version described in this paper is the first version, LAQN00000000.1.
